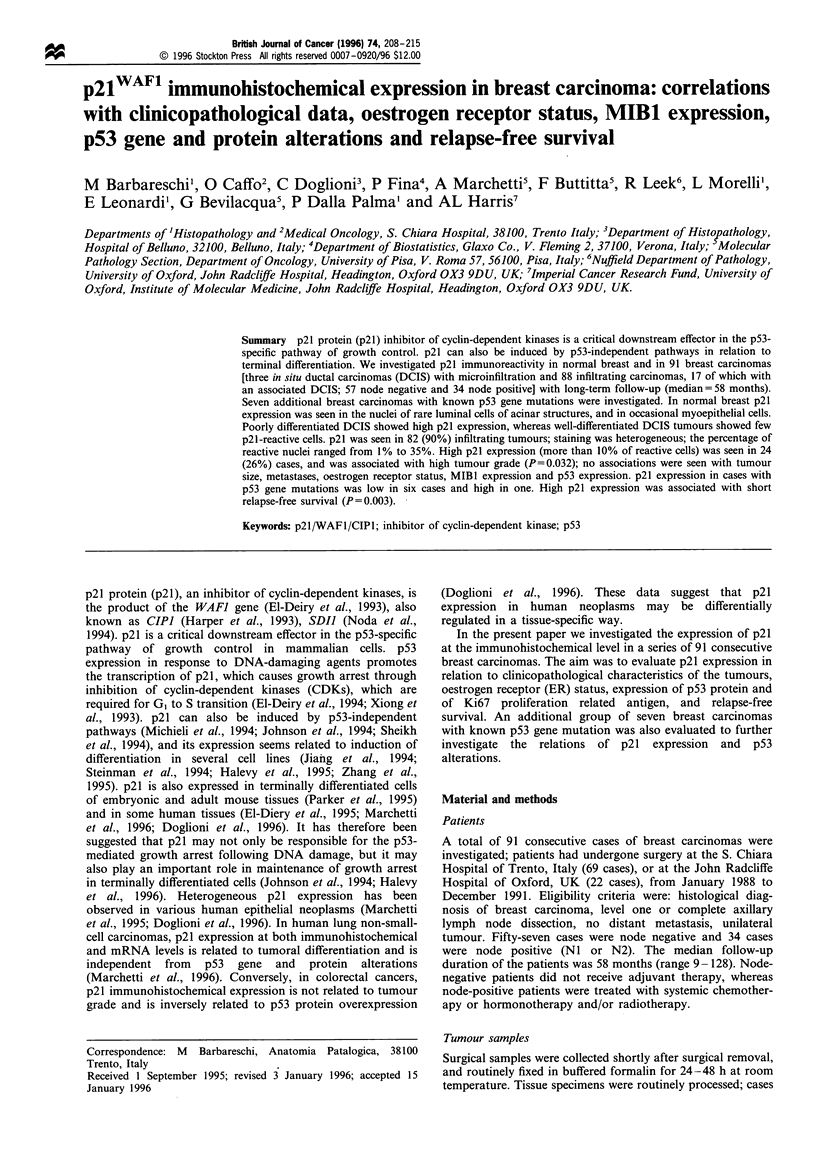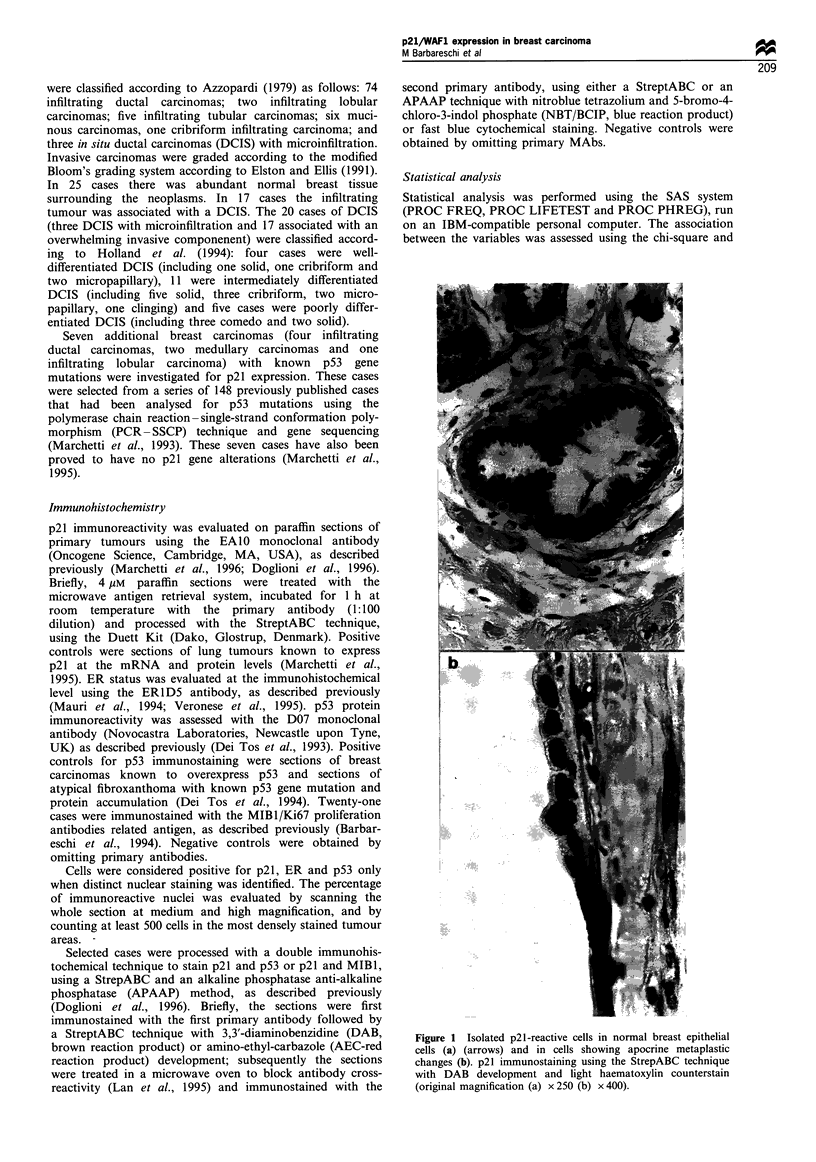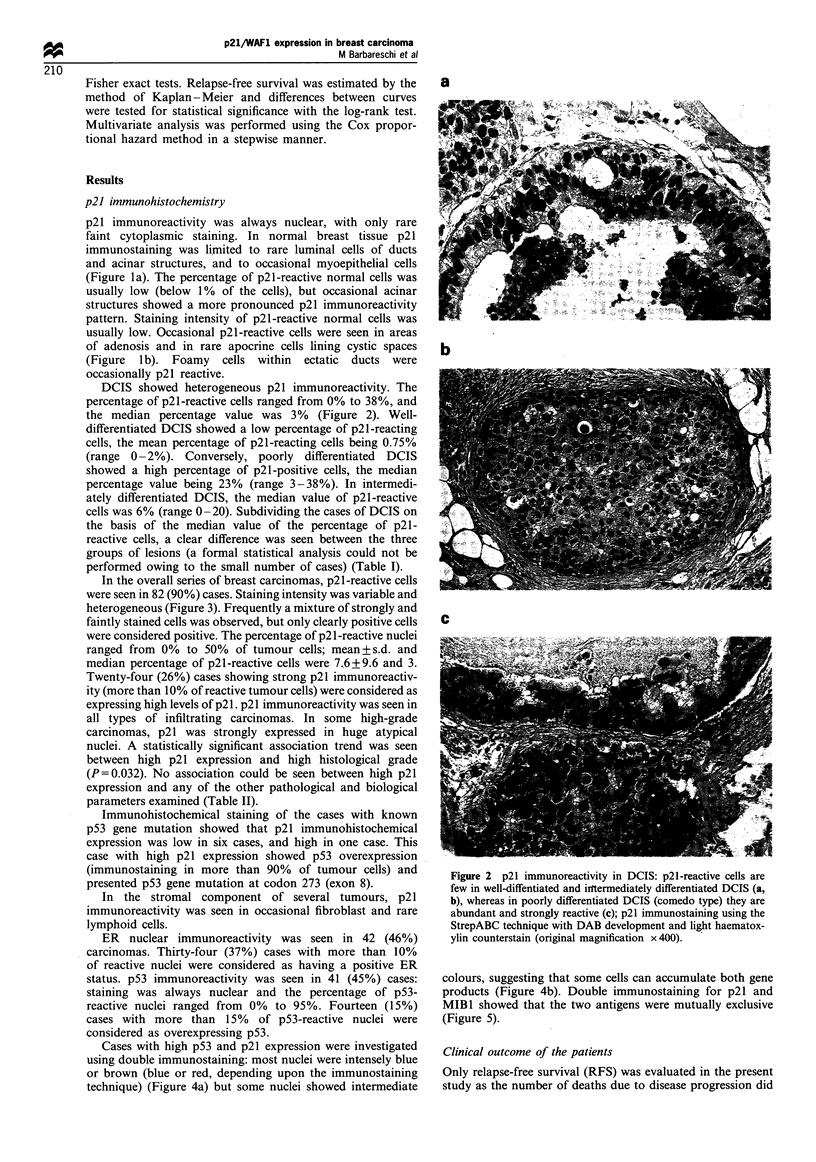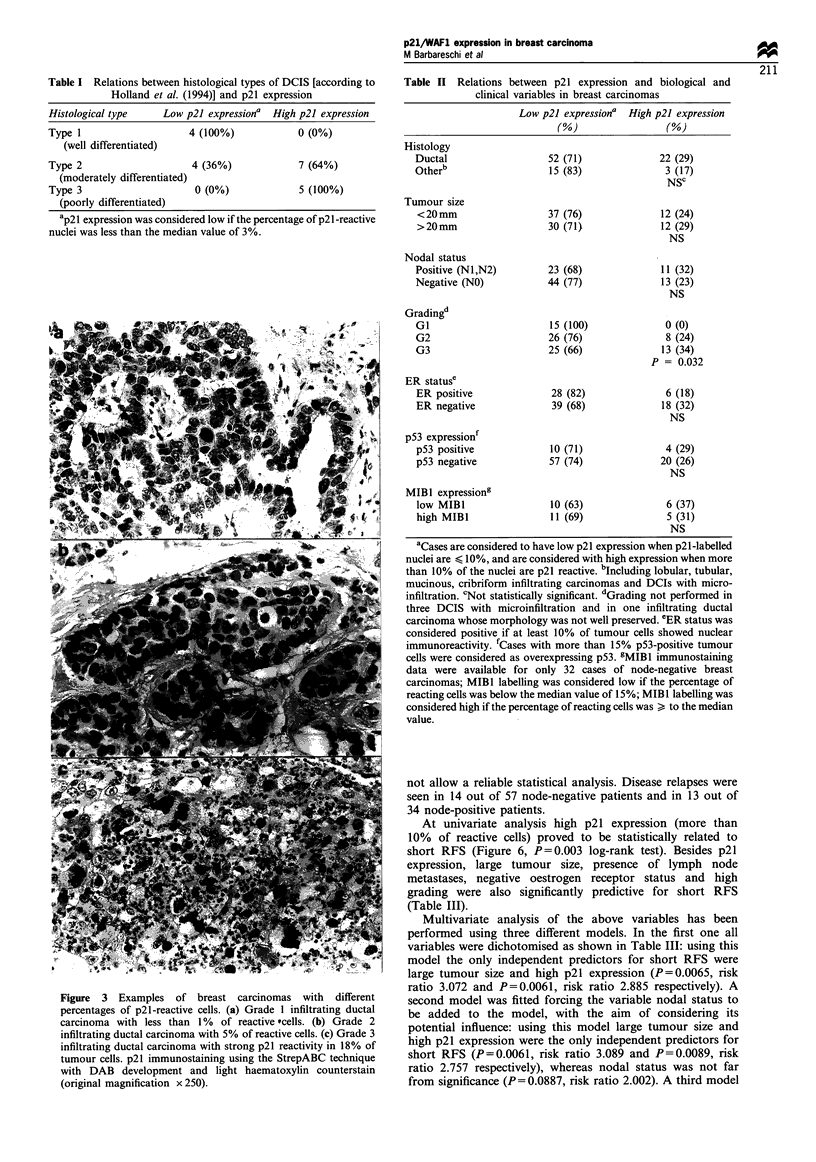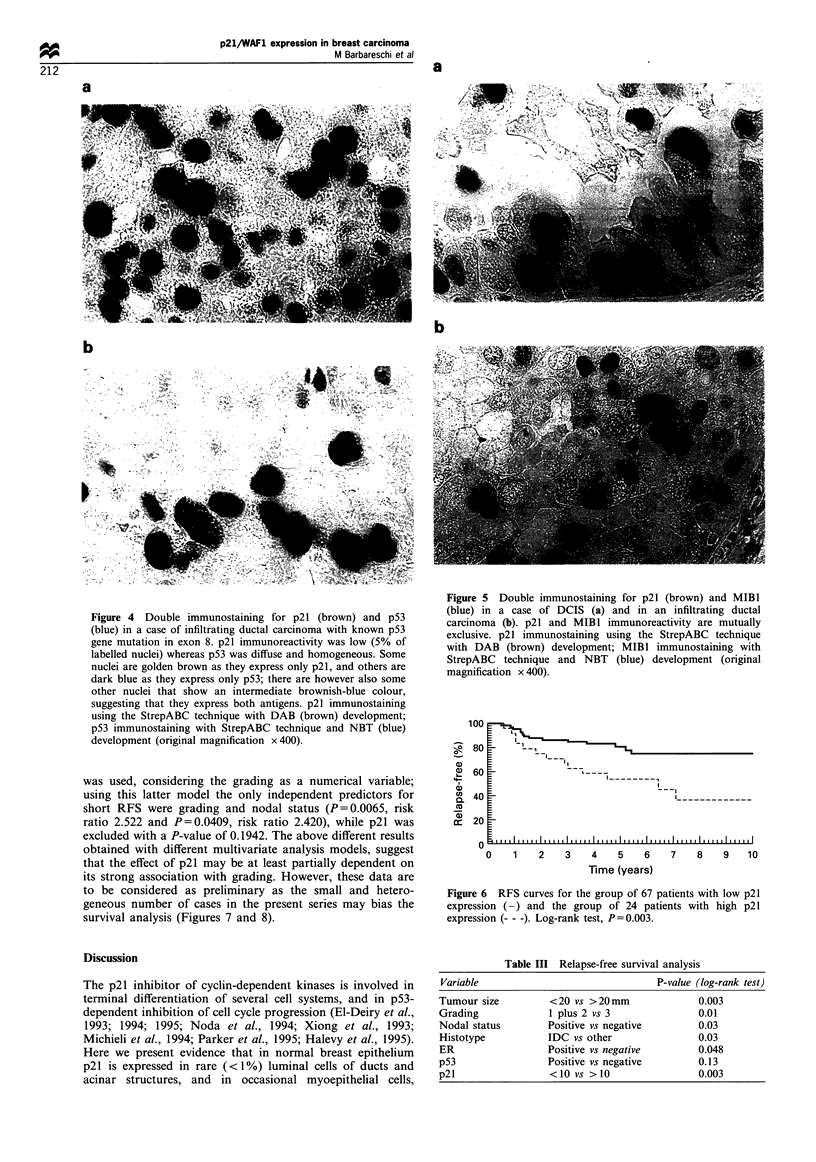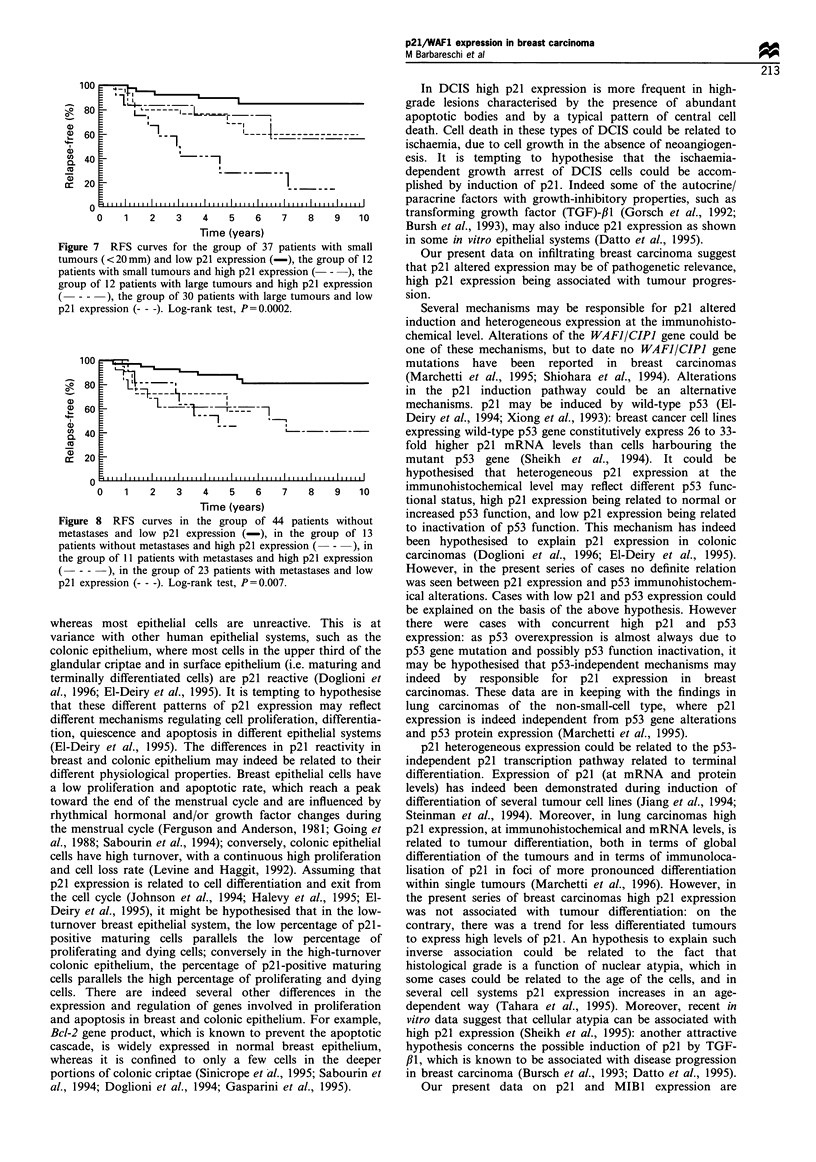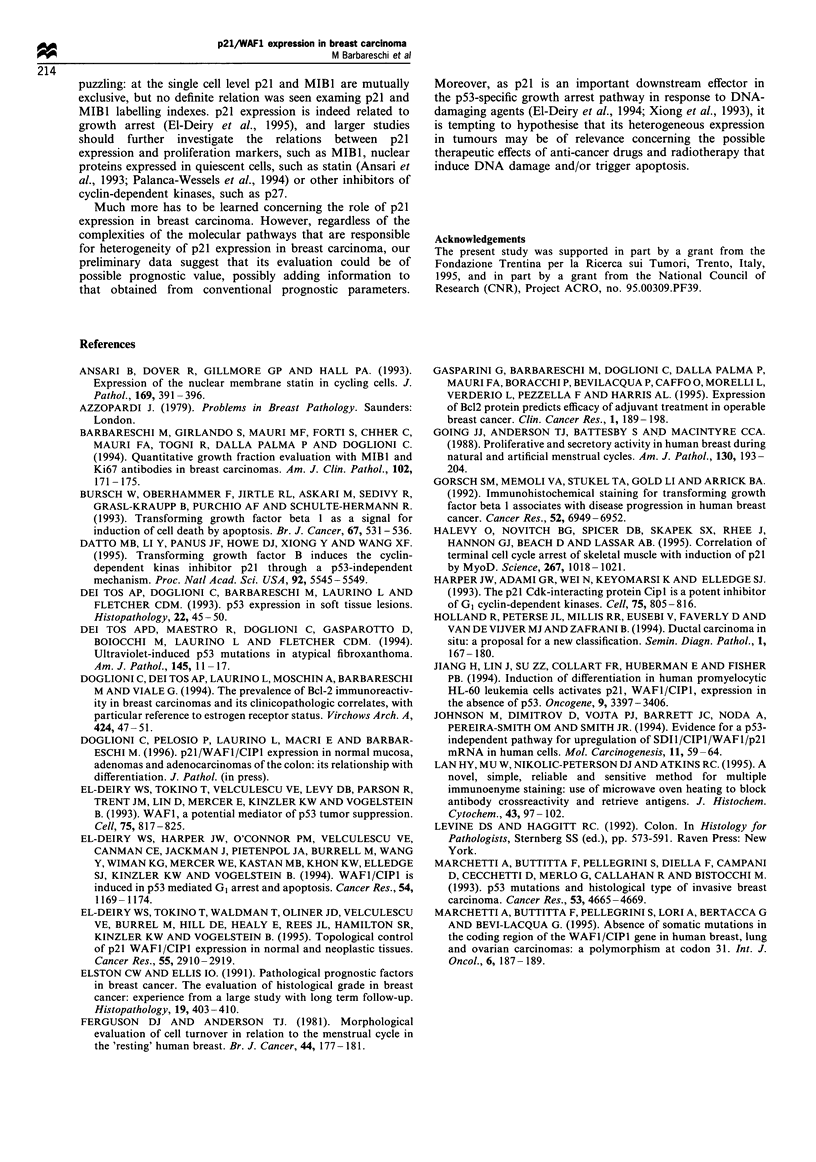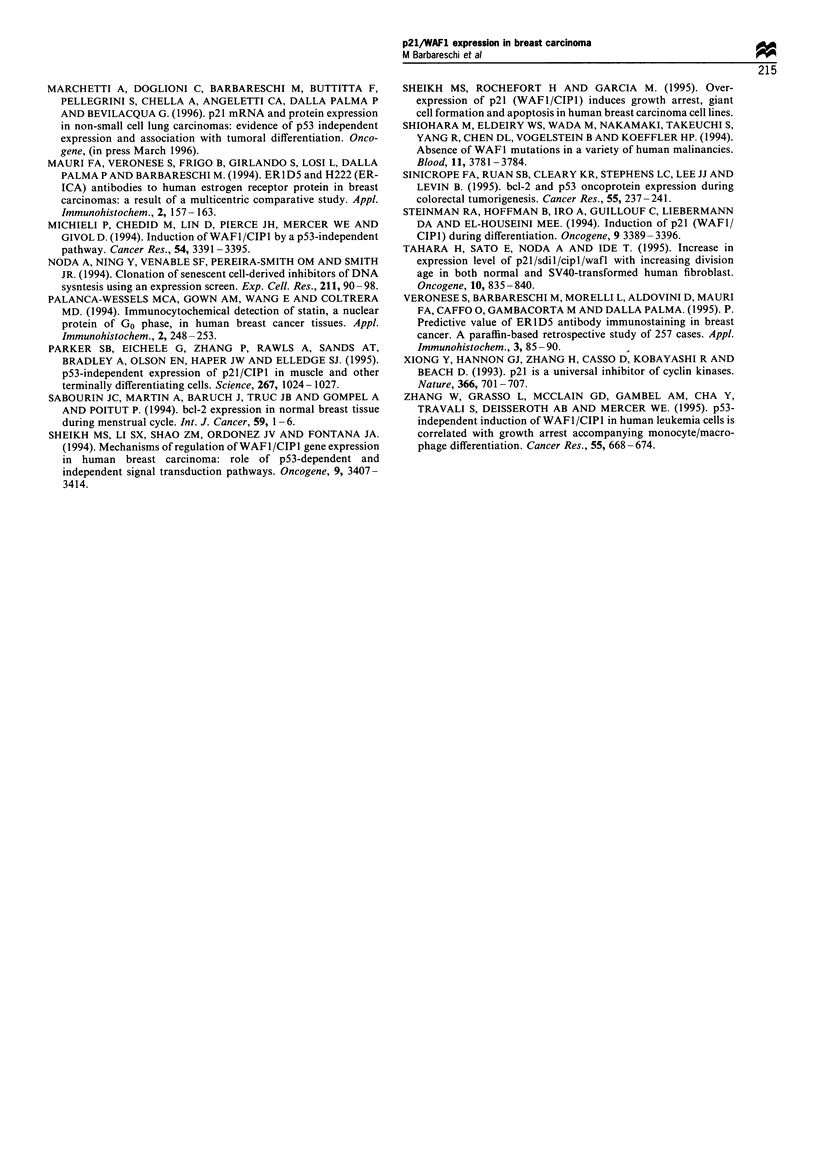# p21WAF1 immunohistochemical expression in breast carcinoma: correlations with clinicopathological data, oestrogen receptor status, MIB1 expression, p53 gene and protein alterations and relapse-free survival.

**DOI:** 10.1038/bjc.1996.339

**Published:** 1996-07

**Authors:** M. Barbareschi, O. Caffo, C. Doglioni, P. Fina, A. Marchetti, F. Buttitta, R. Leek, L. Morelli, E. Leonardi, G. Bevilacqua, P. Dalla Palma, A. L. Harris

**Affiliations:** Department of Histopathology, S. Chiara Hospital, Trento Italy.

## Abstract

**Images:**


British Journal of Cancer (1996) 74, 208-215
© 1996 Stockton Press All rights reserved 0007-0920/96 $12.00

### p21$^{WAF1}$ immunohistochemical expression in breast carcinoma: correlations with clinicopathological data, oestrogen receptor status, MIB1 expression, p53 gene and protein alterations and relapse-free survival

M Barbareschi[1], O Caffo[2], C Doglioni[3], P Fina[4], A Marchetti[5], F Buttitta[5], R Leek[6], L Morelli[1], E Leonardi[1], G Bevilacqua[5], P Dalla Palma[1] and AL Harris[7]

*Departments of [1]Histopathology and [2]Medical Oncology, S. Chiara Hospital, 38100, Trento Italy; [3]Department of Histopathology, Hospital of Belluno, 32100, Belluno, Italy; [4]Department of Biostatistics, Glaxo Co., V. Fleming 2, 37100, Verona, Italy; [5]Molecular Pathology Section, Department of Oncology, University of Pisa, V. Roma 57, 56100, Pisa, Italy; [6]Nuffield Department of Pathology, University of Oxford, John Radcliffe Hospital, Headington, Oxford OX3 9DU, UK; [7]Imperial Cancer Research Fund, University of Oxford, Institute of Molecular Medicine, John Radcliffe Hospital, Headington, Oxford OX3 9DU, UK.*

**Summary** p21 protein (p21) inhibitor of cyclin-dependent kinases is a critical downstream effector in the p53-specific pathway of growth control. p21 can also be induced by p53-independent pathways in relation to terminal differentiation. We investigated p21 immunoreactivity in normal breast and in 91 breast carcinomas [three *in situ* ductal carcinomas (DCIS) with microinfiltration and 88 infiltrating carcinomas, 17 of which with an associated DCIS; 57 node negative and 34 node positive] with long-term follow-up (median = 58 months). Seven additional breast carcinomas with known p53 gene mutations were investigated. In normal breast p21 expression was seen in the nuclei of rare luminal cells of acinar structures, and in occasional myoepithelial cells. Poorly differentiated DCIS showed high p21 expression, whereas well-differentiated DCIS tumours showed few p21-reactive cells. p21 was seen in 82 (90%) infiltrating tumours; staining was heterogeneous; the percentage of reactive nuclei ranged from 1% to 35%. High p21 expression (more than 10% of reactive cells) was seen in 24 (26%) cases, and was associated with high tumour grade ($P = 0.032$); no associations were seen with tumour size, metastases, oestrogen receptor status, MIB1 expression and p53 expression. p21 expression in cases with p53 gene mutations was low in six cases and high in one. High p21 expression was associated with short relapse-free survival ($P = 0.003$).

**Keywords:** p21/WAF1/CIP1; inhibitor of cyclin-dependent kinase; p53

p21 protein (p21), an inhibitor of cyclin-dependent kinases, is the product of the *WAF1* gene (El-Deiry *et al.*, 1993), also known as *CIP1* (Harper *et al.*, 1993), *SDI1* (Noda *et al.*, 1994). p21 is a critical downstream effector in the p53-specific pathway of growth control in mammalian cells. p53 expression in response to DNA-damaging agents promotes the transcription of p21, which causes growth arrest through inhibition of cyclin-dependent kinases (CDKs), which are required for $G_1$ to S transition (El-Deiry *et al.*, 1994; Xiong *et al.*, 1993). p21 can also be induced by p53-independent pathways (Michieli *et al.*, 1994; Johnson *et al.*, 1994; Sheikh *et al.*, 1994), and its expression seems related to induction of differentiation in several cell lines (Jiang *et al.*, 1994; Steinman *et al.*, 1994; Halevy *et al.*, 1995; Zhang *et al.*, 1995). p21 is also expressed in terminally differentiated cells of embryonic and adult mouse tissues (Parker *et al.*, 1995) and in some human tissues (El-Diery *et al.*, 1995; Marchetti *et al.*, 1996; Doglioni *et al.*, 1996). It has therefore been suggested that p21 may not only be responsible for the p53-mediated growth arrest following DNA damage, but it may also play an important role in maintenance of growth arrest in terminally differentiated cells (Johnson *et al.*, 1994; Halevy *et al.*, 1996). Heterogeneous p21 expression has been observed in various human epithelial neoplasms (Marchetti *et al.*, 1995; Doglioni *et al.*, 1996). In human lung non-small-cell carcinomas, p21 expression at both immunohistochemical and mRNA levels is related to tumoral differentiation and is independent from p53 gene and protein alterations (Marchetti *et al.*, 1996). Conversely, in colorectal cancers, p21 immunohistochemical expression is not related to tumour grade and is inversely related to p53 protein overexpression (Doglioni *et al.*, 1996). These data suggest that p21 expression in human neoplasms may be differentially regulated in a tissue-specific way.

In the present paper we investigated the expression of p21 at the immunohistochemical level in a series of 91 consecutive breast carcinomas. The aim was to evaluate p21 expression in relation to clinicopathological characteristics of the tumours, oestrogen receptor (ER) status, expression of p53 protein and of Ki67 proliferation related antigen, and relapse-free survival. An additional group of seven breast carcinomas with known p53 gene mutation was also evaluated to further investigate the relations of p21 expression and p53 alterations.

### Material and methods

### *Patients*

A total of 91 consecutive cases of breast carcinomas were investigated; patients had undergone surgery at the S. Chiara Hospital of Trento, Italy (69 cases), or at the John Radcliffe Hospital of Oxford, UK (22 cases), from January 1988 to December 1991. Eligibility criteria were: histological diagnosis of breast carcinoma, level one or complete axillary lymph node dissection, no distant metastasis, unilateral tumour. Fifty-seven cases were node negative and 34 cases were node positive (N1 or N2). The median follow-up duration of the patients was 58 months (range 9-128). Node-negative patients did not receive adjuvant therapy, whereas node-positive patients were treated with systemic chemotherapy or hormonotherapy and/or radiotherapy.

### *Tumour samples*

Surgical samples were collected shortly after surgical removal, and routinely fixed in buffered formalin for 24-48 h at room temperature. Tissue specimens were routinely processed; cases

Correspondence: M Barbareschi, Anatomia Patalogica, 38100 Trento, Italy
Received 1 September 1995; revised 3 January 1996; accepted 15 January 1996

were classified according to Azzopardi (1979) as follows: 74 infiltrating ductal carcinomas; two infiltrating lobular carcinomas; five infiltrating tubular carcinomas; six mucinous carcinomas, one cribriform infiltrating carcinoma; and three *in situ* ductal carcinomas (DCIS) with microinfiltration. Invasive carcinomas were graded according to the modified Bloom's grading system according to Elston and Ellis (1991). In 25 cases there was abundant normal breast tissue surrounding the neoplasms. In 17 cases the infiltrating tumour was associated with a DCIS. The 20 cases of DCIS (three DCIS with microinfiltration and 17 associated with an overwhelming invasive componenent) were classified according to Holland *et al.* (1994): four cases were well-differentiated DCIS (including one solid, one cribriform and two micropapillary), 11 were intermediately differentiated DCIS (including five solid, three cribriform, two micropapillary, one clinging) and five cases were poorly differentiated DCIS (including three comedo and two solid).

Seven additional breast carcinomas (four infiltrating ductal carcinomas, two medullary carcinomas and one infiltrating lobular carcinoma) with known p53 gene mutations were investigated for p21 expression. These cases were selected from a series of 148 previously published cases that had been analysed for p53 mutations using the polymerase chain reaction – single-strand conformation polymorphism (PCR – SSCP) technique and gene sequencing (Marchetti *et al.*, 1993). These seven cases have also been proved to have no p21 gene alterations (Marchetti *et al.*, 1995).

*Immunohistochemistry*

p21 immunoreactivity was evaluated on paraffin sections of primary tumours using the EA10 monoclonal antibody (Oncogene Science, Cambridge, MA, USA), as described previously (Marchetti *et al.*, 1996; Doglioni *et al.*, 1996). Briefly, 4 $\mu$M paraffin sections were treated with the microwave antigen retrieval system, incubated for 1 h at room temperature with the primary antibody (1:100 dilution) and processed with the StreptABC technique, using the Duett Kit (Dako, Glostrup, Denmark). Positive controls were sections of lung tumours known to express p21 at the mRNA and protein levels (Marchetti *et al.*, 1995). ER status was evaluated at the immunohistochemical level using the ER1D5 antibody, as described previously (Mauri *et al.*, 1994; Veronese *et al.*, 1995). p53 protein immunoreactivity was assessed with the D07 monoclonal antibody (Novocastra Laboratories, Newcastle upon Tyne, UK) as described previously (Dei Tos *et al.*, 1993). Positive controls for p53 immunostaining were sections of breast carcinomas known to overexpress p53 and sections of atypical fibroxanthoma with known p53 gene mutation and protein accumulation (Dei Tos *et al.*, 1994). Twenty-one cases were immunostained with the MIB1/Ki67 proliferation antibodies related antigen, as described previously (Barbareschi *et al.*, 1994). Negative controls were obtained by omitting primary antibodies.

Cells were considered positive for p21, ER and p53 only when distinct nuclear staining was identified. The percentage of immunoreactive nuclei was evaluated by scanning the whole section at medium and high magnification, and by counting at least 500 cells in the most densely stained tumour areas.

Selected cases were processed with a double immunohistochemical technique to stain p21 and p53 or p21 and MIB1, using a StrepABC and an alkaline phosphatase anti-alkaline phosphatase (APAAP) method, as described previously (Doglioni *et al.*, 1996). Briefly, the sections were first immunostained with the first primary antibody followed by a StreptABC technique with 3,3′-diaminobenzidine (DAB, brown reaction product) or amino-ethyl-carbazole (AEC-red reaction product) development; subsequently the sections were treated in a microwave oven to block antibody cross-reactivity (Lan *et al.*, 1995) and immunostained with the second primary antibody, using either a StreptABC or an APAAP technique with nitroblue tetrazolium and 5-bromo-4-chloro-3-indol phosphate (NBT/BCIP, blue reaction product) or fast blue cytochemical staining. Negative controls were obtained by omitting primary MAbs.

*Statistical analysis*

Statistical analysis was performed using the SAS system (PROC FREQ, PROC LIFETEST and PROC PHREG), run on an IBM-compatible personal computer. The association between the variables was assessed using the chi-square and

**Figure 1** Isolated p21-reactive cells in normal breast epithelial cells (**a**) (arrows) and in cells showing apocrine metaplastic changes (**b**). p21 immunostaining using the StrepABC technique with DAB development and light haematoxylin counterstain (original magnification (a) ×250 (b) ×400).

Fisher exact tests. Relapse-free survival was estimated by the method of Kaplan–Meier and differences between curves were tested for statistical significance with the log-rank test. Multivariate analysis was performed using the Cox proportional hazard method in a stepwise manner.

### Results

### *p21 immunohistochemistry*

p21 immunoreactivity was always nuclear, with only rare faint cytoplasmic staining. In normal breast tissue p21 immunostaining was limited to rare luminal cells of ducts and acinar structures, and to occasional myoepithelial cells (Figure 1a). The percentage of p21-reactive normal cells was usually low (below 1% of the cells), but occasional acinar structures showed a more pronounced p21 immunoreactivity pattern. Staining intensity of p21-reactive normal cells was usually low. Occasional p21-reactive cells were seen in areas of adenosis and in rare apocrine cells lining cystic spaces (Figure 1b). Foamy cells within ectatic ducts were occasionally p21 reactive.

DCIS showed heterogeneous p21 immunoreactivity. The percentage of p21-reactive cells ranged from 0% to 38%, and the median percentage value was 3% (Figure 2). Well-differentiated DCIS showed a low percentage of p21-reacting cells, the mean percentage of p21-reacting cells being 0.75% (range 0–2%). Conversely, poorly differentiated DCIS showed a high percentage of p21-positive cells, the median percentage value being 23% (range 3–38%). In intermediately differentiated DCIS, the median value of p21-reactive cells was 6% (range 0–20). Subdividing the cases of DCIS on the basis of the median value of the percentage of p21-reactive cells, a clear difference was seen between the three groups of lesions (a formal statistical analysis could not be performed owing to the small number of cases) (Table I).

In the overall series of breast carcinomas, p21-reactive cells were seen in 82 (90%) cases. Staining intensity was variable and heterogeneous (Figure 3). Frequently a mixture of strongly and faintly stained cells was observed, but only clearly positive cells were considered positive. The percentage of p21-reactive nuclei ranged from 0% to 50% of tumour cells; mean±s.d. and median percentage of p21-reactive cells were 7.6±9.6 and 3. Twenty-four (26%) cases showing strong p21 immunoreactivity (more than 10% of reactive tumour cells) were considered as expressing high levels of p21. p21 immunoreactivity was seen in all types of infiltrating carcinomas. In some high-grade carcinomas, p21 was strongly expressed in huge atypical nuclei. A statistically significant association trend was seen between high p21 expression and high histological grade ($P=0.032$). No association could be seen between high p21 expression and any of the other pathological and biological parameters examined (Table II).

Immunohistochemical staining of the cases with known p53 gene mutation showed that p21 immunohistochemical expression was low in six cases, and high in one case. This case with high p21 expression showed p53 overexpression (immunostaining in more than 90% of tumour cells) and presented p53 gene mutation at codon 273 (exon 8).

In the stromal component of several tumours, p21 immunoreactivity was seen in occasional fibroblast and rare lymphoid cells.

ER nuclear immunoreactivity was seen in 42 (46%) carcinomas. Thirty-four (37%) cases with more than 10% of reactive nuclei were considered as having a positive ER status. p53 immunoreactivity was seen in 41 (45%) cases: staining was always nuclear and the percentage of p53-reactive nuclei ranged from 0% to 95%. Fourteen (15%) cases with more than 15% of p53-reactive nuclei were considered as overexpressing p53.

Cases with high p53 and p21 expression were investigated using double immunostaining: most nuclei were intensely blue or brown (blue or red, depending upon the immunostaining technique) (Figure 4a) but some nuclei showed intermediate colours, suggesting that some cells can accumulate both gene products (Figure 4b). Double immunostaining for p21 and MIB1 showed that the two antigens were mutually exclusive (Figure 5).

**Figure 2** p21 immunoreactivity in DCIS: p21-reactive cells are few in well-diffentiated and intermediately differentiated DCIS (**a**, **b**), whereas in poorly differentiated DCIS (comedo type) they are abundant and strongly reactive (**c**); p21 immunostaining using the StrepABC technique with DAB development and light haematoxylin counterstain (original magnification ×400).

### *Clinical outcome of the patients*

Only relapse-free survival (RFS) was evaluated in the present study as the number of deaths due to disease progression did

**Table I** Relations between histological types of DCIS [according to Holland *et al.* (1994)] and p21 expression

| *Histological type* | *Low p21 expression*[a] | *High p21 expression* |
|---|---|---|
| Type 1 (well differentiated) | 4 (100%) | 0 (0%) |
| Type 2 (moderately differentiated) | 4 (36%) | 7 (64%) |
| Type 3 (poorly differentiated) | 0 (0%) | 5 (100%) |

[a]p21 expression was considered low if the percentage of p21-reactive nuclei was less than the median value of 3%.

**Figure 3** Examples of breast carcinomas with different percentages of p21-reactive cells. (**a**) Grade 1 infiltrating ductal carcinoma with less than 1% of reactive cells. (**b**) Grade 2 infiltrating ductal carcinoma with 5% of reactive cells. (**c**) Grade 3 infiltrating ductal carcinoma with strong p21 reactivity in 18% of tumour cells. p21 immunostaining using the StrepABC technique with DAB development and light haematoxylin counterstain (original magnification ×250).

**Table II** Relations between p21 expression and biological and clinical variables in breast carcinomas

| | *Low p21 expression*[a] *(%)* | *High p21 expression* *(%)* |
|---|---|---|
| Histology | | |
| Ductal | 52 (71) | 22 (29) |
| Other[b] | 15 (83) | 3 (17) |
| | | NS[c] |
| Tumour size | | |
| <20 mm | 37 (76) | 12 (24) |
| >20 mm | 30 (71) | 12 (29) |
| | | NS |
| Nodal status | | |
| Positive (N1,N2) | 23 (68) | 11 (32) |
| Negative (N0) | 44 (77) | 13 (23) |
| | | NS |
| Grading[d] | | |
| G1 | 15 (100) | 0 (0) |
| G2 | 26 (76) | 8 (24) |
| G3 | 25 (66) | 13 (34) |
| | | $P = 0.032$ |
| ER status[e] | | |
| ER positive | 28 (82) | 6 (18) |
| ER negative | 39 (68) | 18 (32) |
| | | NS |
| p53 expression[f] | | |
| p53 positive | 10 (71) | 4 (29) |
| p53 negative | 57 (74) | 20 (26) |
| | | NS |
| MIB1 expression[g] | | |
| low MIB1 | 10 (63) | 6 (37) |
| high MIB1 | 11 (69) | 5 (31) |
| | | NS |

[a]Cases are considered to have low p21 expression when p21-labelled nuclei are ⩽10%, and are considered with high expression when more than 10% of the nuclei are p21 reactive. [b]Including lobular, tubular, mucinous, cribriform infiltrating carcinomas and DCIs with micro-infiltration. [c]Not statistically significant. [d]Grading not performed in three DCIS with microinfiltration and in one infiltrating ductal carcinoma whose morphology was not well preserved. [e]ER status was considered positive if at least 10% of tumour cells showed nuclear immunoreactivity. [f]Cases with more than 15% p53-positive tumour cells were considered as overexpressing p53. [g]MIB1 immunostaining data were available for only 32 cases of node-negative breast carcinomas; MIB1 labelling was considered low if the percentage of reacting cells was below the median value of 15%; MIB1 labelling was considered high if the percentage of reacting cells was ⩾ to the median value.

not allow a reliable statistical analysis. Disease relapses were seen in 14 out of 57 node-negative patients and in 13 out of 34 node-positive patients.

At univariate analysis high p21 expression (more than 10% of reactive cells) proved to be statistically related to short RFS (Figure 6, $P = 0.003$ log-rank test). Besides p21 expression, large tumour size, presence of lymph node metastases, negative oestrogen receptor status and high grading were also significantly predictive for short RFS (Table III).

Multivariate analysis of the above variables has been performed using three different models. In the first one all variables were dichotomised as shown in Table III: using this model the only independent predictors for short RFS were large tumour size and high p21 expression ($P = 0.0065$, risk ratio 3.072 and $P = 0.0061$, risk ratio 2.885 respectively). A second model was fitted forcing the variable nodal status to be added to the model, with the aim of considering its potential influence: using this model large tumour size and high p21 expression were the only independent predictors for short RFS ($P = 0.0061$, risk ratio 3.089 and $P = 0.0089$, risk ratio 2.757 respectively), whereas nodal status was not far from significance ($P = 0.0887$, risk ratio 2.002). A third model

**Figure 4** Double immunostaining for p21 (brown) and p53 (blue) in a case of infiltrating ductal carcinoma with known p53 gene mutation in exon 8. p21 immunoreactivity was low (5% of labelled nuclei) whereas p53 was diffuse and homogeneous. Some nuclei are golden brown as they express only p21, and others are dark blue as they express only p53; there are however also some other nuclei that show an intermediate brownish-blue colour, suggesting that they express both antigens. p21 immunostaining using the StrepABC technique with DAB (brown) development; p53 immunostaining with StrepABC technique and NBT (blue) development (original magnification ×400).

was used, considering the grading as a numerical variable; using this latter model the only independent predictors for short RFS were grading and nodal status ($P=0.0065$, risk ratio 2.522 and $P=0.0409$, risk ratio 2.420), while p21 was excluded with a $P$-value of 0.1942. The above different results obtained with different multivariate analysis models, suggest that the effect of p21 may be at least partially dependent on its strong association with grading. However, these data are to be considered as preliminary as the small and heterogeneous number of cases in the present series may bias the survival analysis (Figures 7 and 8).

### Discussion

The p21 inhibitor of cyclin-dependent kinases is involved in terminal differentiation of several cell systems, and in p53-dependent inhibition of cell cycle progression (El-Deiry *et al.*, 1993; 1994; 1995; Noda *et al.*, 1994; Xiong *et al.*, 1993; Michieli *et al.*, 1994; Parker *et al.*, 1995; Halevy *et al.*, 1995). Here we present evidence that in normal breast epithelium p21 is expressed in rare (<1%) luminal cells of ducts and acinar structures, and in occasional myoepithelial cells,

**Figure 5** Double immunostaining for p21 (brown) and MIB1 (blue) in a case of DCIS (**a**) and in an infiltrating ductal carcinoma (**b**). p21 and MIB1 immunoreactivity are mutually exclusive. p21 immunostaining using the StrepABC technique with DAB (brown) development; MIB1 immunostaining with StrepABC technique and NBT (blue) development (original magnification ×400).

**Figure 6** RFS curves for the group of 67 patients with low p21 expression (–) and the group of 24 patients with high p21 expression (- - -). Log-rank test, $P=0.003$.

**Table III** Relapse-free survival analysis

| *Variable* | | P-*value (log-rank test)* |
|---|---|---|
| Tumour size | <20 *vs* >20mm | 0.003 |
| Grading | 1 plus 2 *vs* 3 | 0.01 |
| Nodal status | Positive *vs* negative | 0.03 |
| Histotype | IDC *vs* other | 0.03 |
| ER | Positive *vs negative* | 0.048 |
| p53 | Positive *vs* negative | 0.13 |
| p21 | <10 *vs* >10 | 0.003 |

**Figure 7** RFS curves for the group of 37 patients with small tumours (<20 mm) and low p21 expression (—), the group of 12 patients with small tumours and high p21 expression (— - —), the group of 12 patients with large tumours and high p21 expression (— - - - —), the group of 30 patients with large tumours and low p21 expression (- - -). Log-rank test, $P=0.0002$.

**Figure 8** RFS curves in the group of 44 patients without metastases and low p21 expression (—), in the group of 13 patients without metastases and high p21 expression (— - —), in the group of 11 patients with metastases and high p21 expression (— - - - —), in the group of 23 patients with metastases and low p21 expression (- - -). Log-rank test, $P=0.007$.

whereas most epithelial cells are unreactive. This is at variance with other human epithelial systems, such as the colonic epithelium, where most cells in the upper third of the glandular criptae and in surface epithelium (i.e. maturing and terminally differentiated cells) are p21 reactive (Doglioni *et al.*, 1996; El-Deiry *et al.*, 1995). It is tempting to hypothesise that these different patterns of p21 expression may reflect different mechanisms regulating cell proliferation, differentiation, quiescence and apoptosis in different epithelial systems (El-Deiry *et al.*, 1995). The differences in p21 reactivity in breast and colonic epithelium may indeed be related to their different physiological properties. Breast epithelial cells have a low proliferation and apoptotic rate, which reach a peak toward the end of the menstrual cycle and are influenced by rhythmical hormonal and/or growth factor changes during the menstrual cycle (Ferguson and Anderson, 1981; Going *et al.*, 1988; Sabourin *et al.*, 1994); conversely, colonic epithelial cells have high turnover, with a continuous high proliferation and cell loss rate (Levine and Haggit, 1992). Assuming that p21 expression is related to cell differentiation and exit from the cell cycle (Johnson *et al.*, 1994; Halevy *et al.*, 1995; El-Deiry *et al.*, 1995), it might be hypothesised that in the low-turnover breast epithelial system, the low percentage of p21-positive maturing cells parallels the low percentage of proliferating and dying cells; conversely in the high-turnover colonic epithelium, the percentage of p21-positive maturing cells parallels the high percentage of proliferating and dying cells. There are indeed several other differences in the expression and regulation of genes involved in proliferation and apoptosis in breast and colonic epithelium. For example, *Bcl-2* gene product, which is known to prevent the apoptotic cascade, is widely expressed in normal breast epithelium, whereas it is confined to only a few cells in the deeper portions of colonic criptae (Sinicrope *et al.*, 1995; Sabourin *et al.*, 1994; Doglioni *et al.*, 1994; Gasparini *et al.*, 1995).

In DCIS high p21 expression is more frequent in high-grade lesions characterised by the presence of abundant apoptotic bodies and by a typical pattern of central cell death. Cell death in these types of DCIS could be related to ischaemia, due to cell growth in the absence of neoangiogenesis. It is tempting to hypothesise that the ischaemia-dependent growth arrest of DCIS cells could be accomplished by induction of p21. Indeed some of the autocrine/paracrine factors with growth-inhibitory properties, such as transforming growth factor (TGF)-$\beta 1$ (Gorsch *et al.*, 1992; Bursh *et al.*, 1993), may also induce p21 expression as shown in some *in vitro* epithelial systems (Datto *et al.*, 1995).

Our present data on infiltrating breast carcinoma suggest that p21 altered expression may be of pathogenetic relevance, high p21 expression being associated with tumour progression.

Several mechanisms may be responsible for p21 altered induction and heterogeneous expression at the immunohistochemical level. Alterations of the *WAF1/CIP1* gene could be one of these mechanisms, but to date no *WAF1/CIP1* gene mutations have been reported in breast carcinomas (Marchetti *et al.*, 1995; Shiohara *et al.*, 1994). Alterations in the p21 induction pathway could be an alternative mechanisms. p21 may be induced by wild-type p53 (El-Deiry *et al.*, 1994; Xiong *et al.*, 1993): breast cancer cell lines expressing wild-type p53 gene constitutively express 26 to 33-fold higher p21 mRNA levels than cells harbouring the mutant p53 gene (Sheikh *et al.*, 1994). It could be hypothesised that heterogeneous p21 expression at the immunohistochemical level may reflect different p53 functional status, high p21 expression being related to normal or increased p53 function, and low p21 expression being related to inactivation of p53 function. This mechanism has indeed been hypothesised to explain p21 expression in colonic carcinomas (Doglioni *et al.*, 1996; El-Deiry *et al.*, 1995). However, in the present series of cases no definite relation was seen between p21 expression and p53 immunohistochemical alterations. Cases with low p21 and p53 expression could be explained on the basis of the above hypothesis. However there were cases with concurrent high p21 and p53 expression: as p53 overexpression is almost always due to p53 gene mutation and possibly p53 function inactivation, it may be hypothesised that p53-independent mechanisms may indeed by responsible for p21 expression in breast carcinomas. These data are in keeping with the findings in lung carcinomas of the non-small-cell type, where p21 expression is indeed independent from p53 gene alterations and p53 protein expression (Marchetti *et al.*, 1995).

p21 heterogeneous expression could be related to the p53-independent p21 transcription pathway related to terminal differentiation. Expression of p21 (at mRNA and protein levels) has indeed been demonstrated during induction of differentiation of several tumour cell lines (Jiang *et al.*, 1994; Steinman *et al.*, 1994). Moreover, in lung carcinomas high p21 expression, at immunohistochemical and mRNA levels, is related to tumour differentiation, both in terms of global differentiation of the tumours and in terms of immunolocalisation of p21 in foci of more pronounced differentiation within single tumours (Marchetti *et al.*, 1996). However, in the present series of breast carcinomas high p21 expression was not associated with tumour differentiation: on the contrary, there was a trend for less differentiated tumours to express high levels of p21. An hypothesis to explain such inverse association could be related to the fact that histological grade is a function of nuclear atypia, which in some cases could be related to the age of the cells, and in several cell systems p21 expression increases in an age-dependent way (Tahara *et al.*, 1995). Moreover, recent *in vitro* data suggest that cellular atypia can be associated with high p21 expression (Sheikh *et al.*, 1995): another attractive hypothesis concerns the possible induction of p21 by TGF-$\beta 1$, which is known to be associated with disease progression in breast carcinoma (Bursch *et al.*, 1993; Datto *et al.*, 1995).

Our present data on p21 and MIB1 expression are puzzling: at the single cell level p21 and MIB1 are mutually exclusive, but no definite relation was seen examing p21 and MIB1 labelling indexes. p21 expression is indeed related to growth arrest (El-Deiry *et al.*, 1995), and larger studies should further investigate the relations between p21 expression and proliferation markers, such as MIB1, nuclear proteins expressed in quiescent cells, such as statin (Ansari *et al.*, 1993; Palanca-Wessels *et al.*, 1994) or other inhibitors of cyclin-dependent kinases, such as p27.

Much more has to be learned concerning the role of p21 expression in breast carcinoma. However, regardless of the complexities of the molecular pathways that are responsible for heterogeneity of p21 expression in breast carcinoma, our preliminary data suggest that its evaluation could be of possible prognostic value, possibly adding information to that obtained from conventional prognostic parameters. Moreover, as p21 is an important downstream effector in the p53-specific growth arrest pathway in response to DNA-damaging agents (El-Deiry *et al.*, 1994; Xiong *et al.*, 1993), it is tempting to hypothesise that its heterogeneous expression in tumours may be of relevance concerning the possible therapeutic effects of anti-cancer drugs and radiotherapy that induce DNA damage and/or trigger apoptosis.

**Acknowledgements**
The present study was supported in part by a grant from the Fondazione Trentina per la Ricerca sui Tumori, Trento, Italy, 1995, and in part by a grant from the National Council of Research (CNR), Project ACRO, no. 95.00309.PF39.

### References

ANSARI B, DOVER R, GILLMORE GP AND HALL PA. (1993). Expression of the nuclear membrane statin in cycling cells. *J. Pathol.*, **169**, 391–396.

AZZOPARDI J. (1979). *Problems in Breast Pathology*. Saunders: London.

BARBARESCHI M, GIRLANDO S, MAURI MF, FORTI S, CHHER C, MAURI FA, TOGNI R, DALLA PALMA P AND DOGLIONI C. (1994). Quantitative growth fraction evaluation with MIB1 and Ki67 antibodies in breast carcinomas. *Am. J. Clin. Pathol.*, **102**, 171–175.

BURSCH W, OBERHAMMER F, JIRTLE RL, ASKARI M, SEDIVY R, GRASL-KRAUPP B, PURCHIO AF AND SCHULTE-HERMANN R. (1993). Transforming growth factor beta 1 as a signal for induction of cell death by apoptosis. *Br. J. Cancer*, **67**, 531–536.

DATTO MB, LI Y, PANUS JF, HOWE DJ, XIONG Y AND WANG XF. (1995). Transforming growth factor B induces the cyclin-dependent kinas inhibitor p21 through a p53-independent mechanism. *Proc. Natl Acad. Sci. USA*, **92**, 5545–5549.

DEI TOS AP, DOGLIONI C, BARBARESCHI M, LAURINO L AND FLETCHER CDM. (1993). p53 expression in soft tissue lesions. *Histopathology*, **22**, 45–50.

DEI TOS APD, MAESTRO R, DOGLIONI C, GASPAROTTO D, BOIOCCHI M, LAURINO L AND FLETCHER CDM. (1994). Ultraviolet-induced p53 mutations in atypical fibroxanthoma. *Am. J. Pathol.*, **145**, 11–17.

DOGLIONI C, DEI TOS AP, LAURINO L, MOSCHIN A, BARBARESCHI M AND VIALE G. (1994). The prevalence of Bcl-2 immunoreactivity in breast carcinomas and its clinicopathologic correlates, with particular reference to estrogen receptor status. *Virchows Arch. A*, **424**, 47–51.

DOGLIONI C, PELOSIO P, LAURINO L, MACRI E AND BARBARESCHI M. (1996). p21/WAF1/CIP1 expression in normal mucosa, adenomas and adenocarcinomas of the colon: its relationship with differentiation. *J. Pathol.* (in press).

EL-DEIRY WS, TOKINO T, VELCULESCU VE, LEVY DB, PARSON R, TRENT JM, LIN D, MERCER E, KINZLER KW AND VOGELSTEIN B. (1993). WAF1, a potential mediator of p53 tumor suppression. *Cell*, **75**, 817–825.

EL-DEIRY WS, HARPER JW, O'CONNOR PM, VELCULESCU VE, CANMAN CE, JACKMAN J, PIETENPOL JA, BURRELL M, WANG Y, WIMAN KG, MERCER WE, KASTAN MB, KHON KW, ELLEDGE SJ, KINZLER KW AND VOGELSTEIN B. (1994). WAF1/CIP1 is induced in p53 mediated $G_1$ arrest and apoptosis. *Cancer Res.*, **54**, 1169–1174.

EL-DEIRY WS, TOKINO T, WALDMAN T, OLINER JD, VELCULESCU VE, BURREL M, HILL DE, HEALY E, REES JL, HAMILTON SR, KINZLER KW AND VOGELSTEIN B. (1995). Topological control of p21 WAF1/CIP1 expression in normal and neoplastic tissues. *Cancer Res.*, **55**, 2910–2919.

ELSTON CW AND ELLIS IO. (1991). Pathological prognostic factors in breast cancer. The evaluation of histological grade in breast cancer: experience from a large study with long term follow-up. *Histopathology*, **19**, 403–410.

FERGUSON DJ AND ANDERSON TJ. (1981). Morphological evaluation of cell turnover in relation to the menstrual cycle in the 'resting' human breast. *Br. J. Cancer*, **44**, 177–181.

GASPARINI G, BARBARESCHI M, DOGLIONI C, DALLA PALMA P, MAURI FA, BORACCHI P, BEVILACQUA P, CAFFO O, MORELLI L, VERDERIO L, PEZZELLA F AND HARRIS AL. (1995). Expression of Bcl2 protein predicts efficacy of adjuvant treatment in operable breast cancer. *Clin. Cancer Res.*, **1**, 189–198.

GOING JJ, ANDERSON TJ, BATTESBY S AND MACINTYRE CCA. (1988). Proliferative and secretory activity in human breast during natural and artificial menstrual cycles. *Am. J. Pathol.*, **130**, 193–204.

GORSCH SM, MEMOLI VA, STUKEL TA, GOLD LI AND ARRICK BA. (1992). Immunohistochemical staining for transforming growth factor beta 1 associates with disease progression in human breast cancer. *Cancer Res.*, **52**, 6949–6952.

HALEVY O, NOVITCH BG, SPICER DB, SKAPEK SX, RHEE J, HANNON GJ, BEACH D AND LASSAR AB. (1995). Correlation of terminal cell cycle arrest of skeletal muscle with induction of p21 by MyoD. *Science*, **267**, 1018–1021.

HARPER JW, ADAMI GR, WEI N, KEYOMARSI K AND ELLEDGE SJ. (1993). The p21 Cdk-interacting protein Cip1 is a potent inhibitor of $G_1$ cyclin-dependent kinases. *Cell*, **75**, 805–816.

HOLLAND R, PETERSE JL, MILLIS RR, EUSEBI V, FAVERLY D AND VAN DE VIJVER MJ AND ZAFRANI B. (1994). Ductal carcinoma in situ: a proposal for a new classification. *Semin. Diagn. Pathol.*, **1**, 167–180.

JIANG H, LIN J, SU ZZ, COLLART FR, HUBERMAN E AND FISHER PB. (1994). Induction of differentiation in human promyelocytic HL-60 leukemia cells activates p21, WAF1/CIP1, expression in the absence of p53. *Oncogene*, **9**, 3397–3406.

JOHNSON M, DIMITROV D, VOJTA PJ, BARRETT JC, NODA A, PEREIRA-SMITH OM AND SMITH JR. (1994). Evidence for a p53-independent pathway for upregulation of SDI1/CIP1/WAF1/p21 mRNA in human cells. *Mol. Carcinogenesis*, **11**, 59–64.

LAN HY, MU W, NIKOLIC-PETERSON DJ AND ATKINS RC. (1995). A novel, simple, reliable and sensitive method for multiple immunoenyme staining: use of microwave oven heating to block antibody crossreactivity and retrieve antigens. *J. Histochem. Cytochem.*, **43**, 97–102.

LEVINE DS AND HAGGITT RC. (1992). Colon. In *Histology for Pathologists*, Sternberg SS (ed.), pp. 573-591. Raven Press: New York.

MARCHETTI A, BUTTITTA F, PELLEGRINI S, DIELLA F, CAMPANI D, CECCHETTI D, MERLO G, CALLAHAN R AND BISTOCCHI M. (1993). p53 mutations and histological type of invasive breast carcinoma. *Cancer Res.*, **53**, 4665–4669.

MARCHETTI A, BUTTITTA F, PELLEGRINI S, LORI A, BERTACCA G AND BEVI-LACQUA G. (1995). Absence of somatic mutations in the coding region of the WAF1/CIP1 gene in human breast, lung and ovarian carcinomas: a polymorphism at codon 31. *Int. J. Oncol.*, **6**, 187–189.

MARCHETTI A, DOGLIONI C, BARBARESCHI M, BUTTITTA F, PELLEGRINI S, CHELLA A, ANGELETTI CA, DALLA PALMA P AND BEVILACQUA G. (1996). p21 mRNA and protein expression in non-small cell lung carcinomas: evidence of p53 independent expression and association with tumoral differentiation. *Oncogene*, (in press March 1996).

MAURI FA, VERONESE S, FRIGO B, GIRLANDO S, LOSI L, DALLA PALMA P AND BARBARESCHI M. (1994). ER1D5 and H222 (ER-ICA) antibodies to human estrogen receptor protein in breast carcinomas: a result of a multicentric comparative study. *Appl. Immunohistochem.*, **2**, 157–163.

MICHIELI P, CHEDID M, LIN D, PIERCE JH, MERCER WE AND GIVOL D. (1994). Induction of WAF1/CIP1 by a p53-independent pathway. *Cancer Res.*, **54**, 3391–3395.

NODA A, NING Y, VENABLE SF, PEREIRA-SMITH OM AND SMITH JR. (1994). Clonation of senescent cell-derived inhibitors of DNA sysntesis using an expression screen. *Exp. Cell. Res.*, **211**, 90–98.

PALANCA-WESSELS MCA, GOWN AM, WANG E AND COLTRERA MD. (1994). Immunocytochemical detection of statin, a nuclear protein of $G_0$ phase, in human breast cancer tissues. *Appl. Immunohistochem.*, **2**, 248–253.

PARKER SB, EICHELE G, ZHANG P, RAWLS A, SANDS AT, BRADLEY A, OLSON EN, HAPER JW AND ELLEDGE SJ. (1995). p53-independent expression of p21/CIP1 in muscle and other terminally differentiating cells. *Science*, **267**, 1024–1027.

SABOURIN JC, MARTIN A, BARUCH J, TRUC JB AND GOMPEL A AND POITUT P. (1994). bcl-2 expression in normal breast tissue during menstrual cycle. *Int. J. Cancer*, **59**, 1–6.

SHEIKH MS, LI SX, SHAO ZM, ORDONEZ JV AND FONTANA JA. (1994). Mechanisms of regulation of WAF1/CIP1 gene expression in human breast carcinoma: role of p53-dependent and independent signal transduction pathways. *Oncogene*, **9**, 3407–3414.

SHEIKH MS, ROCHEFORT H AND GARCIA M. (1995). Overexpression of p21 (WAF1/CIP1) induces growth arrest, giant cell formation and apoptosis in human breast carcinoma cell lines.

SHIOHARA M, ELDEIRY WS, WADA M, NAKAMAKI, TAKEUCHI S, YANG R, CHEN DL, VOGELSTEIN B AND KOEFFLER HP. (1994). Absence of WAF1 mutations in a variety of human malinancies. *Blood*, **11**, 3781–3784.

SINICROPE FA, RUAN SB, CLEARY KR, STEPHENS LC, LEE JJ AND LEVIN B. (1995). bcl-2 and p53 oncoprotein expression during colorectal tumorigenesis. *Cancer Res.*, **55**, 237–241.

STEINMAN RA, HOFFMAN B, IRO A, GUILLOUF C, LIEBERMANN DA AND EL-HOUSEINI MEE. (1994). Induction of p21 (WAF1/CIP1) during differentiation. *Oncogene*, **9** 3389–3396.

TAHARA H, SATO E, NODA A AND IDE T. (1995). Increase in expression level of p21/sdi1/cip1/waf1 with increasing division age in both normal and SV40-transformed human fibroblast. *Oncogene*, **10**, 835–840.

VERONESE S, BARBARESCHI M, MORELLI L, ALDOVINI D, MAURI FA, CAFFO O, GAMBACORTA M AND DALLA PALMA. (1995). P. Predictive value of ER1D5 antibody immunostaining in breast cancer. A paraffin-based retrospective study of 257 cases. *Appl. Immunohistochem.*, **3**, 85–90.

XIONG Y, HANNON GJ, ZHANG H, CASSO D, KOBAYASHI R AND BEACH D. (1993). p21 is a universal inhibitor of cyclin kinases. *Nature*, **366**, 701–707.

ZHANG W, GRASSO L, MCCLAIN GD, GAMBEL AM, CHA Y, TRAVALI S, DEISSEROTH AB AND MERCER WE. (1995). p53-independent induction of WAF1/CIP1 in human leukemia cells is correlated with growth arrest accompanying monocyte/macrophage differentiation. *Cancer Res.*, **55**, 668–674.